# Associations among total and food additive phosphorus intake and carotid intima-media thickness – a cross-sectional study in a middle-aged population in Southern Finland

**DOI:** 10.1186/1475-2891-12-94

**Published:** 2013-07-10

**Authors:** Suvi T Itkonen, Heini J Karp, Virpi E Kemi, Elina M Kokkonen, Elisa M Saarnio, Minna H Pekkinen, Merja UM Kärkkäinen, E Kalevi A Laitinen, Maila I Turanlahti, Christel JE Lamberg-Allardt

**Affiliations:** 1 Department of Food and Environmental Sciences Calcium Research Unit, University of Helsinki, P.O. Box 66, Helsinki 00014, Finland; 2 Department of Food and Environmental Sciences, University of Helsinki, Helsinki, Finland; 3 Department of Medical Genetics, Folkhälsan Institute of Genetics, University of Helsinki, Helsinki, Finland; 4 Department of Obstetrics and Gynecology, Helsinki University Central Hospital, Helsinki, Finland; 5 Department of Pediatric Cardiology, Children’s Hospital, Helsinki University Central Hospital and University of Helsinki, Helsinki, Finland

**Keywords:** Phosphorus, Phosphate, Carotid intima-media thickness, Cardiovascular risk factors

## Abstract

**Background:**

Dietary phosphorus (P) intake in Western countries is 2- to 3-fold higher than recommended, and phosphate is widely used as a food additive in eg. cola beverages and processed meat products. Elevated serum phosphate concentrations have been associated with cardiovascular disease (CVD) risk factors and CVD itself in several studies in patients with renal dysfunction and in a few studies in the general population. Carotid intima-media thickness (IMT) is a CVD risk factor, thus the aim of the study was to determine if an association between dietary P, especially food additive phosphate (FAP), intake, and IMT exists.

**Methods:**

Associations among total phosphorus (TP) and FAP intake and carotid IMT were investigated in a cross-sectional study of 37- to 47-year-old females (n = 370) and males (n = 176) in Finland. Associations among TP intake, FAP intake, and IMT were tested by analysis of covariance (ANCOVA) in quintiles (TP) and sextiles (FAP) using sex, age, low-density/high-density lipoprotein cholesterol ratio, smoking status, and IMT sonographer as covariates.

**Results:**

No significant associations were present between TP or FAP intake and IMT (p > 0.05, ANCOVA), but in between-group comparisons some differences were found indicating higher IMT among subjects with higher P intake. When testing for a significant linear trend with contrast analysis, a positive trend was observed between energy-adjusted TP intake and IMT among all subjects (p = 0.039), and among females a tendency for a trend existed (p = 0.067). Among all subjects, a significant positive linear trend was also present between FAP intake and IMT (p = 0.022); this trend was also seen in females (p = 0.045). In males, no significant associations or trends were noted between TP or FAP intake and IMT (p > 0.05).

**Conclusions:**

Our results indicate that a significant linear trend exists between energy-adjusted TP intake and FAP intake, and IMT among all subjects. Based on these results, high dietary P intake should be further investigated due to its potential association with adverse cardiovascular health effects in the general population.

## Introduction

Dietary phosphorus (P) intake in Western countries is 2- to 3-fold higher [1-3] than the nutritional recommendations (600–700 mg/d) [4,5] due to abundant consumption of dairy and meat products. P intake has also increased as a consequence of the expanding use of phosphates as additives in the food industry. In the United States, P intake from additives is estimated to have doubled to 1000 mg/d from 1990; 50% of P intake in Western countries comes from the additives as “hidden phosphorus” [6]. P additives also seem to be more absorbable in the intestine than natural P [7,8].

The detrimental effects of dietary P and elevated serum phosphate (S-Pi) concentrations on cardiovascular disease (CVD) were first perceived in renal patients [9]. In the last decade, results on S-Pi were found also in studies of subjects with normal renal function; associations between S-Pi, mortality, and CVD emerged [10], and elevated S-Pi concentrations even in young persons were associated with atherosclerosis risk [11] and higher carotid intima-media thickness (IMT) [12], which is a CVD risk factor. Only limited data on the direct effects of a high-P diet on cardiovascular health are available. In animals, high-P diet has increased vascular calcification [13] and in humans has impaired endothelial function of the vascular system been reported [14]. However, according to a recent opinion in European Heart Journal, not only S-Pi but also dietary P has been proposed to be considered as a CVD risk factor [15].

No studies focusing on associations between dietary P intake and IMT in the normal population have been carried out. Here, we hypothesize that high P intake, especially food additive phosphate (FAP) intake, is associated with higher IMT. We investigated by a cross-sectional design in a middle-aged Finnish population the associations among total dietary P and FAP intake and IMT to determine whether high dietary P intake should be considered a CVD risk factor in a general population.

## Materials and methods

### Study subjects and study design

**Figure 1:**
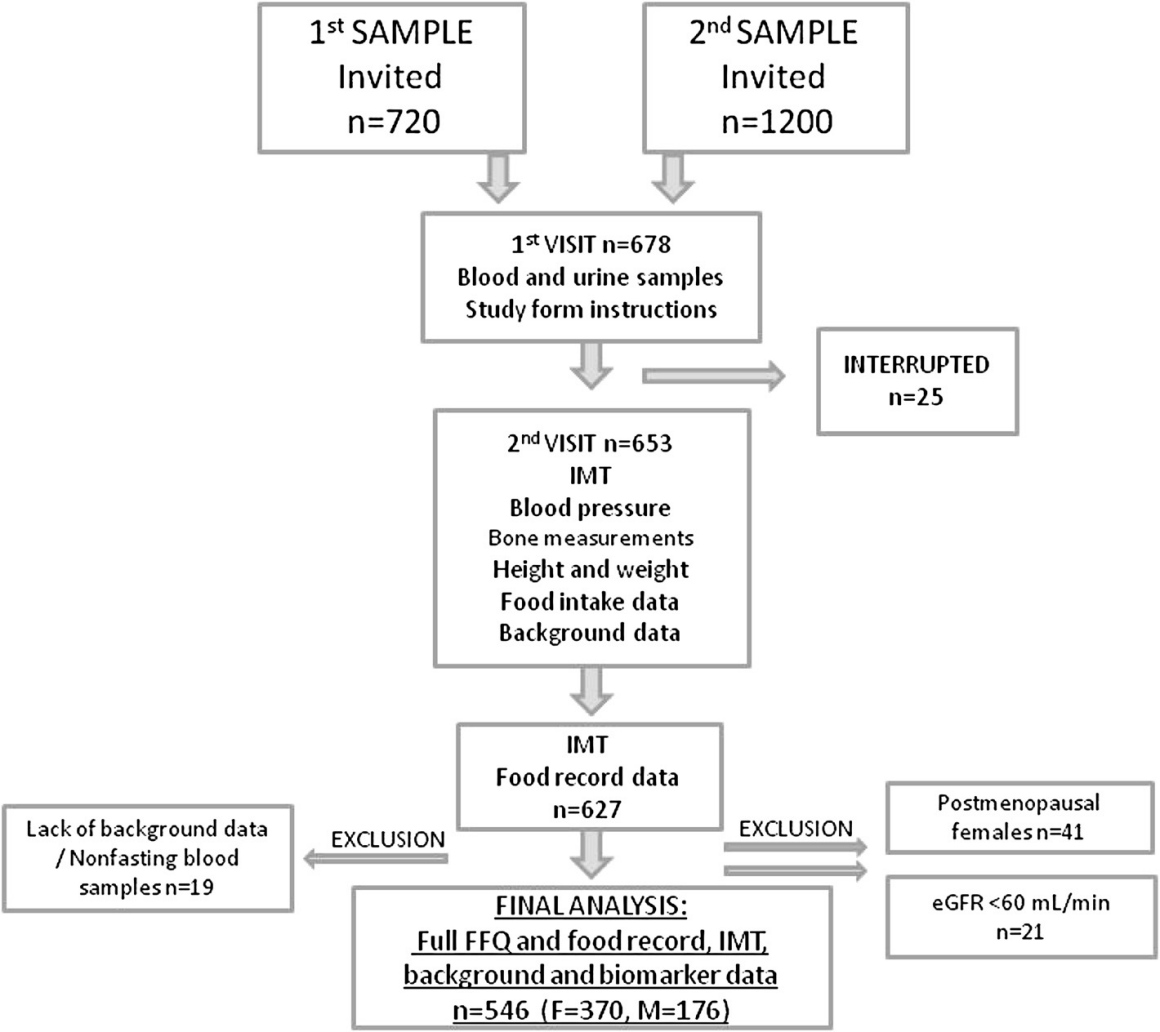
**Sampling and protocol of the study.** IMT = intima-media thickness, eGFR = estimated glomerular filtration rate, FFQ = food frequency questionnaire, F = female, M = male.

A randomly collected sample of 1920 subjects aged 37–47 years (50% males, 50% females) and living in the Helsinki area, was derived from the Population Register Centre in Finland. Subjects were contacted by mail in November 2009 (1st sampling) and February 2010 (2nd sampling) and invited to participate in the study. All subjects gave their informed consent to the procedures, which were in accord with the Helsinki Declaration. The study protocol was approved by Helsinki Uusimaa Hospital District Ethics Committees. The study protocol and sampling are illustrated in Figure 1. The initial exclusion criterion was pregnancy.

Subjects visited the research unit two times during spring 2010: on the first visit (January or March), fasting blood samples and spot urine samples were collected and subjects were advised to fill in the study form, which included food records, food frequency questionnaires (FFQ) concerning phosphorus, calcium, and vitamin D intake, and a background questionnaire. Before the second visit, subjects were advised to abstain from caffeine-containing products and smoking for four hours. During this visit common carotid IMT was scanned by ultrasonography, blood pressure was measured by an automatic device, volumetric bone mineral density and bone geometry were assessed from the tibia and radius by peripheral quantitative computed tomography (results not shown), and subjects’ height and weight were measured. All forms were checked by the researchers, and additional information was requested if needed.

Totally 678 persons participated in the first visit to the research unit either in January or March 2010 and 653 of them in the second visit during spring 2010 (interruption for unknown reasons, n = 25). IMT and food record data were successfully collected from 627 persons. Of them, postmenopausal females (n = 41, data collected by a questionnaire) were excluded from the analyses, and persons with moderate renal dysfunction (estimated glomerular filtration rate, eGFR < 60 ml/min) as well (n = 21) due to potentially altered mineral metabolism. Additionally, 19 persons were excluded because they did not complete the study due to lack of background data or due to non-fasting blood samples. The final analyses included 546 subjects (370 females, 176 males) for whom full nutrition, IMT, background, and biomarker data were available (Figure 1). No significant (p > 0.05) differences were present in age, sex, IMT, or total phosphorus (TP) intake between excluded subjects (n = 81) and the included ones. Some differences did, however, emerge in body mass index (BMI) (p < 0.001) and pulse pressure (p = 0.018); included subjects were heavier and had higher pulse pressure than the excluded ones.

### IMT and blood pressure measurements

Common carotid artery IMT was measured by a semi-automatic measurement program using high-resolution ultrasonography (Esaote MyLab30Gold, Firenze, Italy). IMT was measured from a 1-cm sample from the bifurcation (bulb) with an 8- or 10-mHz linear array transducer by three experienced sonographers. The software measured the far wall thickness bilaterally for the 1-cm segment. The measurement was carried out on the left and right carotid in duplicate with the subject in a supine position [16]. The mean intima-media complex thickness was automatically calculated in μm in diastolic phase scans. The averages of these four measurements were used in the analyses. Repeatability of the measurements was ensured by rescanning 18 participants within one week of the first IMT scanning. In addition, IMT of 15 participants was measured by two sonographers. The coefficient of variation between the measurements obtained by the same sonographer (intra CV%) was 6.6 and between measurements obtained by different sonographers (inter CV%) 9.2. Vascular calcification was not assessed in this study. The systolic and diastolic blood pressures were measured after the IMT measurement in supine position in duplicate by an automatic device (Omron MIT Elite Digital Automatic Blood Pressure Monitor, OMRON Healthcare Europe B.V., Hoofddorp, the Netherlands), and the second result was used in the analyses. Pulse pressure was calculated as systolic – diastolic.

### Biomarkers

Twelve-hour fasting blood samples were collected between 7:30 am and 9:15 am. Serum / plasma was extracted from blood by centrifugation (except whole-blood samples) and stored immediately after sampling at -20°C to -70°C until analysis. Serum total cholesterol, high-density lipoprotein cholesterol (HDL-C), low-density lipoprotein cholesterol (LDL-C), triglycerides, creatinine, glucose, high-sensitivity C-reactive protein, calcium (Ca) and phosphate (Pi) concentrations, and glycosylated hemoglobin (HbA1c%) concentrations from whole-blood samples were analyzed by a photometric method using a Konelab20 automatic analyzer (Thermo Clinical Labsystems, Espoo, Finland). Inter and intra CV%s were <4.6 for the above-mentioned analyses. Serum 25-hydroxy vitamin D (S-25(OH)D) concentrations were analyzed by using an IDS enzyme immunoassay kit (Immunodiagnostics Systems Ltd., Boldon, UK). Inter and intra CV%s were 2.7 and 3.2, respectively. Serum intact parathyroid hormone (S-iPTH) concentrations were analyzed by immunoluminescence-based method by Immulite1000 (Siemens Healthcare Diagnostics, Malvern, PA, USA). Inter and intra CV%s were 8.0 and <5.5, respectively. Serum insulin concentrations were also analyzed by Immulite1000, the inter and intra CV%s being <4.9 and <6.2, respectively. Plasma carboxy-terminal fibroblast growth factor (P-FGF-23) concentrations were evaluated by using an Immutopics enzyme immunoassay kit (Immutopics Inc., San Clemente, CA, USA), and inter and intra CV%s were <5.5 and <7.7, respectively. LDL-C/HDL-C ratio was calculated as LDL-C/HDL-C. eGFR was determined by using the Cockroft-Gault formula [17].

### Dietary intake and background data collection

Habitual dietary intake data of subjects were collected by 3-day food records, which included two weekdays and one weekend day. The subjects were instructed to maintain their normal food habits during the recording period and to record all foods, beverages, and dietary supplements immediately after consumption. Nutrient intake was calculated using a computer-based program (Diet 32 version 1.4.6.2, Aivo2000, Turku, Finland) based on the Finnish food composition database Fineli® (Institution of Health and Welfare, Helsinki, Finland). Approximately 400 new recipes were added to the program to ensure accurate calculation of nutrient intake. Vitamin D and Ca supplementation was taken into account in the calculations.

FAP intake calculations were based on one-month food use frequency data, which were collected by a validated FFQ concerning P intake, ranging from “less than one portion per month” to daily portions [18]. The FFQ included P sources in different food groups, distinguishing FAP-containing foodstuffs from natural P-containing foodstuffs, and also including P sources not consumed every day. For FAP intake calculations, we chose products known to contain FAP and separated these into food groups meeting the EU regulation for maximum FAP content (meat products like marinated meat, sausages, and cold cuts; cola beverages; processed cheeses, Table 1). FAP intake from different sources was calculated from the portions of FAP-containing foodstuffs as grams and recorded on the FFQ, using the maximum amount of FAP allowed in the foodstuff according to European Union regulations (meat products 5 g/kg; cola beverages 700 mg/L; processed cheeses 20 g/kg [19]). We used only these products because in them the amount of added FAP is regulated, unlike for example starch-based FAPs. Cookies were excluded from the calculations because some do not contain FAPs. We divided subjects into tertiles concerning each FAP group (meat products, cola beverages, processed cheeses) intake, with score 0 indicating the lowest intake tertile, score 1 the middle tertile, and score 2 the highest tertile. We summed the scores from different FAP sources for each subject; thus, each person was given a score from zero to six. No-one received a score of 0, thus, we used the scoring 1–6 and divided the subjects into six groups; the final group sizes were sufficient for analysis of covariance (ANCOVA). Due to the method, the sizes of FAP intake groups were not equal. We used this calculation method because the exact FAP content in foodstuffs is unknown. When analyzing the sexes separately, the calculations were done in sex-specific groups.

**Table 1:** Food additive phosphate (FAP) -containing products included in FAP intake calculations

**FAP-containing product**	**Portion size**
**Processed meat products**	
Processed, marinated chicken, turkey, beef,	1 steak (130 g)
pork, or other meat	
Liver pâté and liver sausage, regular and light	1 tablespoon (15 g)
Sausages / frankfurters, regular and light	10 cm slice of big sausage, 3 frankfurters
	or 1 barbeque sausage (100 g)
Meat cold cuts, regular and light	1 slice (15 g)
Sausage-style cold cuts, regular and light	1 slice (15 g)
**Processed cheeses**	
With different fat contents:	1 tablespoon or
>24%, 20-24%, 9-19%, and 9%	one individually packed slice (15 g)
**Cola beverages**	
Regular and light	One glass (200 mL)

Background data were collected by a questionnaire covering disease history and lifestyle factors such as smoking status, physical activity, and menopause. BMI was calculated as weight (kg) / height (m)^2^.

### Statistical analysis

Statistical analysis was performed using PASW Statistics version 18.0.2 (IBM, Armonk, NY, USA). The normality and homogeneity of the data were verified. Equality of the variances in quintiles and FAP groups was assessed by Levene’s test.

Correlations among IMT, TP, energy-adjusted TP (eTP), and FAP intake and potential confounding factors were assessed by Pearson correlation coefficients (data not shown). We chose covariates depending on their correlation with IMT and P intake and their contextual significance. Highly intercorrelated (r > 0.2) confounding factors were not used in the same models. Due to the high correlation between Ca and P intake, Ca was excluded from the covariates. BMI correlated with IMT, TP intake, and almost all potential covariates, and thus, BMI was also excluded from the covariates. We used serum LDL-C/HDL-C ratio because it predicts IMT progression better than HDL-C or LDL-C alone [20]. IMT scanning results of one sonographer differed significantly from the others, thus, sonographer class was coded as a dummy variable (0 for nondiffering sonographer, 1 for differing sonographer) and used as a random factor in ANCOVA. Smoking status was also coded as dummy (0 for never-smoker, 1 for former or current smoker). The final covariates were sex (when analyzing all subjects), age, smoking status, LDL-C/HDL-C ratio, and IMT sonographer as a random factor. We also included pulse pressure in some models, but this did not strengthen the results (data not shown). P value less than 0.05 was considered statistically significant.

Differences between TP / eTP quintiles or FAP groups concerning potential confounding factors were analyzed by analysis of variance (ANOVA, unadjusted for covariates). We used ANCOVA to analyze differences in IMT between TP / eTP / FAP intake quantiles; we were especially interested in determining whether high TP / eTP / FAP intake is associated with higher IMT. Tests for statistically significant linear trends in associations between P variable intakes and IMT were performed by contrast analysis.

## Results

### Characteristics of subjects potentially associated with phosphorus intake

Background and biomarker data of study subjects are shown in Tables 2 and 3, respectively. Below we report the differences (ANOVA, unadjusted for covariates) in the background and biomarker factors described in Tables 2 and 3 in TP and eTP quintiles, and in FAP groups. We report only significant differences between the quantiles for all subjects and also pay attention to S-Pi, P-FGF-23, S-iPTH, and S-25(OH)D concentrations, as well as to Ca:P intake ratios. Results for females were somewhat similar to all subjects, and males had only a few significant differences (data not shown).

**Table 2:** Background data of study subjects

**Variable**	**All (n = 546)**	**Females (n = 370)**	**Males (n = 176)**
Intima-media thickness (μm)	552 ± 69	544 ± 70	567 ± 66
Body mass index (kg/m^2^)	26.8 ± 4.9	26.6 ± 5.2	27.2 ± 3.9
Age (years)	41.9 ± 2.8	41.9 ± 2.7	42.1 ± 3.0
Sex (% females)	67 ± 47	100	-
Current or former smoker (%)	48 ± 50	45 ± 50	55 ± 50
Systolic blood pressure (mmHg)	125 ± 14	122 ± 14	129 ± 13
Diastolic blood pressure (mmHg)	77 ± 11	76 ± 11	80 ± 11
Pulse pressure (mmHg)	48 ± 6	47 ± 6	50 ± 6
All physical activity (mins/week)	479 ± 380	515 ± 397	406 ± 332
Energy intake (kJ/day)	8413 ± 1994	7944 ± 1807	9398 ± 2014
Fat intake (g/day)	80 ± 26	76 ± 26	88 ± 25
Saturated fat intake (g/day)	29 ± 11	28 ± 11	32 ± 11
Phosphorus intake (mg/day)	1617 ± 428	1532 ± 378	1795 ± 469
Calcium intake (mg/day) *	1199 ± 453	1195 ±435	1207 ± 489
Vitamin D intake (ug/day) *	9.1 ± 8.6	9.4 ± 9.3	8.4 ± 6.8
Energy-adjusted phosphorus intake (mg/MJ/day)	194 ± 36	195 ± 37	192 ± 34
FAP intake score	3.2 ± 1.4	3.1 ± 1.3	3.6 ± 1.4

Values are presented as mean ± SD. * supplements included; *FAP* Food additive phosphate.

**Table 3:** Biomarker data of study subjects

**Variable**	**All (n = 546)**	**Females (n = 370)**	**Males (n = 176)**
S-Pi (mmol/L)	1.09 ± 0.15	1.10 ± 0.15	1.06 ± 0.16
S-Ca (mmol/L) ^a^	2.09 ± 0.07	2.09 ± 0.07	2.10 ± 0.07
P-FGF-23, C-terminal (RU/mL)	78.5 ± 84.7	85.1 ± 101.7	64.9 ± 20.1
S-PTH (pg/mL)	55.0 ± 25.2	56.9 ± 26.0	51.0 ± 23.0
S-25(OH)D (nmol/L)	55.4 ± 19.5	56.4 ± 20.0	53.4 ± 18.3
S-total cholesterol (mmol/L)	5.45 ± 0.90	5.29 ± 0.84	5.77 ± 0.93
S-HDL (mmol/L)	1.51 ± 0.40	1.61 ± 0.39	1.33 ± 0.37
S-LDL (mmol/L)	2.94 ± 0.79	2.75 ± 0.72	3.34 ± 0.79
LDL-HDL ratio	2.13 ± 0.94	1.85 ± 0.77	2.71 ± 0.98
S-triglycerides (mmol/L)	1.30 ± 0.73	1.14 ± 0.61	1.62 ± 0.86
eGFR (mL/min)	91.5 ± 20.9	85.6 ± 19.5	103.5 ± 18.6
S-HS-CRP (mg/L)	2.07 ± 4.47	2.13 ± 4.22	1.93 ± 4.97
S-Glucose (mmol/L)	5.08 ± 0.60	5.00 ± 0.48	5.24 ± 0.78
HbA1c%	5.06 ± 0.26	5.04 ± 0.24	5.11 ± 0.28

Values are presented as mean ± SD.

Abbreviation: *S* Serum, *P* Plasma, *Pi* Phosphate *Ca* Calcium *FGF-23* Fibroblast growth factor 23, *PTH* Parathyroid hormone, *25(OH)D* 25-hydroxy vitamin D, *HDL* High-density lipoprotein, *LDL* Low-density lipoprotein, *eGFR* Estimated glomerular filtration rate, *HS-CRP* High-sensitivity C-reactive protein, *HbA1c%* Glycosylated hemoglobin (% of total hemoglobin) ^a^ adjusted to albumin.

#### *Total phosphorus intake*

In TP intake quintiles, the sexes were not distributed equally; the number of females was smaller in higher TP intake quintiles (p < 0.001). LDL-C/HDL-C ratio (p = 0.019), eGFR (p = 0.001), HbA1c% (p = 0.007), energy intake (p < 0.001), total and saturated fat intake (p < 0.001 for both), and Ca and vitamin D intake (p < 0.001) were higher with higher TP intake. No significant differences in S-Pi (p = 0.739), P-FGF-23 (p = 0.511), S-iPTH (p = 0.173), or S-25(OH)D (p = 0.073) concentrations emerged between TP quintiles. Ca:P ratios between quintiles differed almost significantly with a tendency to higher Ca:P ratio with higher TP intake (p = 0.059).

#### *Energy-adjusted total phosphorus intake*

In eTP intake quintiles, energy intake (p < 0.001) and total and saturated fat intake (p < 0.001 for both) were lower when eTP intake was higher. Ca (p < 0.001), vitamin D (p = 0.004), and TP (p < 0.001) intakes were positively related to eTP intake. S-Pi and P-FGF-23 concentrations did not differ significantly across quintiles (p = 0.731 and p = 0.187, respectively). S-iPTH concentrations were lower (p = 0.045) and S-25(OH)D concentrations higher (p = 0.004) with higher eTP intake. Ca:P ratios were lower with lower eTP intake (p = 0.018).

#### *Food additive phosphate intake*

In FAP intake groups, the sexes were not distributed equally; the proportion of females was smaller with higher FAP intake scores (p = 0.003). Differences in BMI were also perceived (p < 0.001); BMI was higher in the highest intake groups. We found differences in pulse pressure (p = 0.019), rising with higher FAP intake. LDL-C/HDL-C ratio (p < 0.001), serum triglycerides (p = 0.003), eGFR (p < 0.001), HbA1c% (p = 0.017), TP (p = 0.015), and energy (p = 0.010) intake were also associated with higher FAP intake. No significant differences emerged in P-FGF-23 (p = 0.499), S-iPTH (p = 0.313), S-25(OH)D concentrations (p = 0.418), or in Ca:P ratios (p = 0.266), but S-Pi concentrations were negatively associated with FAP intake (p = 0.006).

### Associations between phosphorus intake variables and IMT

**Figure 2:**
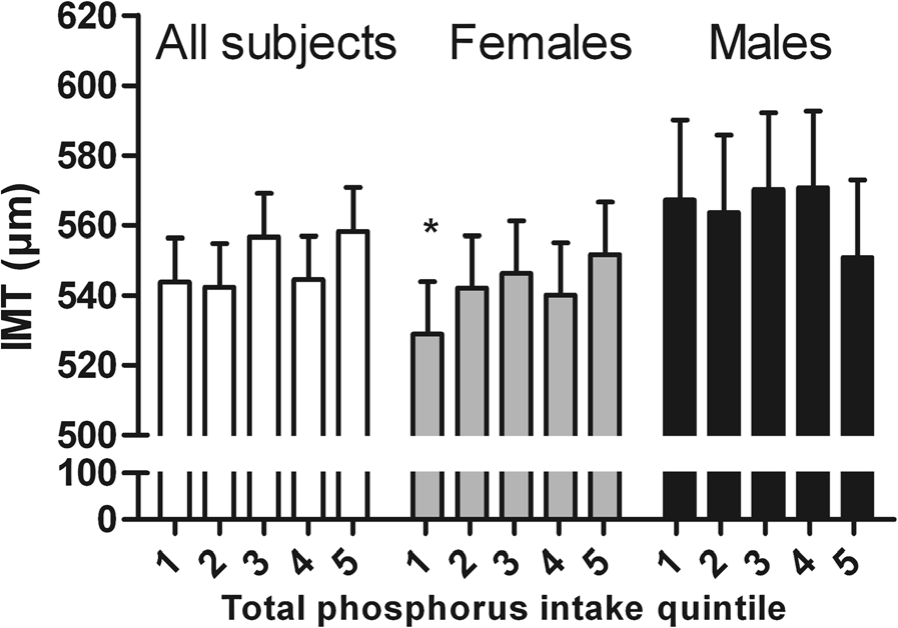
**Mean carotid intima-media thickness (IMT) with upper limit of 95% confidence interval in TOTAL PHOSPHORUS (TP) intake quintiles for ALL SUBJECTS (WHITE SCALE; 1st: 0–1272 mg/d, n = 109; 2nd: 1273–1462 mg/d, n = 109; 3rd: 1463–1665 mg/d, n = 109; 4th: 1666–1962 mg/d, n = 109; 5th: 1963–3205 mg/d, n = 110; p for all 0.233, ANCOVA), for FEMALES (GRAY SCALE; 1st: 0–1232.7 mg/d, n = 74; 2nd: 1233–1390 mg/d, n = 74; 3rd: 1391–1563 mg/d, n = 75; 4th: 1564–1795 mg/d, n = 73; 5th: 1796–2800 mg/d, n = 74; p for all 0.293, ANCOVA) and for MALES (BLACK SCALE; 1st: 0–1443 mg/d, n = 34; 2nd: 1444–1610 mg/d, n = 35; 3rd: 1611–1850 mg/d, n = 35; 4th: 1851–2163.9 mg/d, n = 36; 5th: 2164.0-3205 mg/d, n = 35; p for all 0.719, ANCOVA).** The data are adjusted for sex (when analyzing all subjects), age, LDL-HDL cholesterol ratio, smoking status, and IMT sonographer class. Statistically significant differences from the highest TP intake quintile (ANCOVA) are shown with an asterisk (* p < 0.05) (between all other groups p > 0.05).

**Figure 3:**
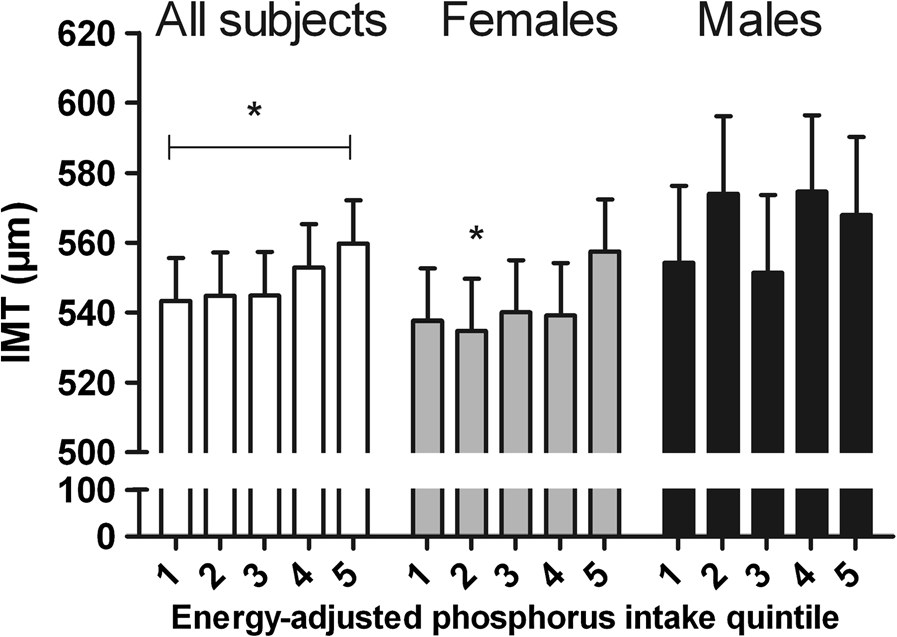
**Mean carotid intima-media thickness (IMT) with upper limit of 95% confidence interval in ENERGY-ADJUSTED PHOSPHORUS (eTP) intake quintiles for ALL SUBJECTS (WHITE SCALE; 1st: 0–162.1 mg/MJ/d, n = 109; 2nd: 162.2-182.8 mg/MJ/d, n = 109; 3rd: 182.9-200.85 mg/MJ/d, n = 109; 4th: 200.86-222.1 mg/MJ/d, n = 109; 5th: 222.2-320 mg/MJ/d, n = 110; p for all 0.289, ANCOVA), for FEMALES (GRAY SCALE; 1st: 0–164.5 mg/MJ/d, n = 74; 2nd: 164.6-183.0 mg/MJ/d, n = 74; 3rd: 183.1-203.0 mg/MJ/d, n = 75; 4th: 203.1-224.0 mg/MJ/d, n = 73; 5th: 224.1-310 mg/MJ/d, n = 74; p for all 0.231, ANCOVA), and for MALES (BLACK SCALE; 1st: 0–160 mg/MJ/d, n = 35; 2nd: 161–182 mg/MJ/d, n = 35; 3rd: 183–198 mg/MJ/d, n = 35; 4th: 199–218.0 mg/MJ/d n = 36; 5th: 218.1-320 mg/MJ/d, n = 35; p for all 0.432, ANCOVA).** The data are adjusted for sex (when analyzing all subjects), age, LDL-HDL cholesterol ratio, smoking status, and IMT sonographer class. Significant differences from the highest eTP intake quintile (ANCOVA) / statistically significant trends (contrast analysis) between eTP intake and IMT are indicated with asterisks (*p < 0.05) (between all other groups p > 0.05).

**Figure 4:**
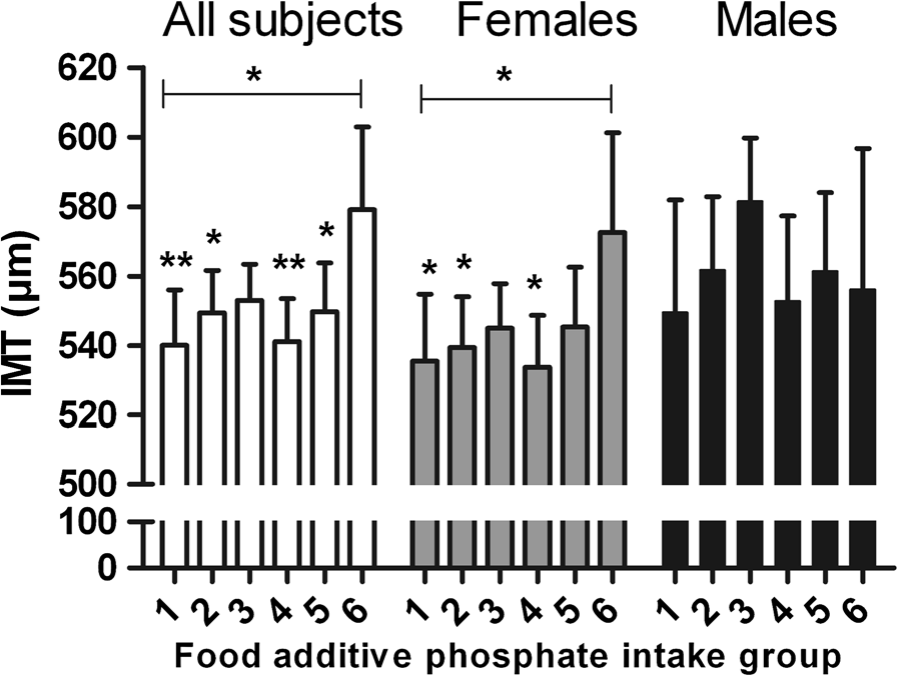
**Mean carotid intima-media thickness (IMT) with upper limit of 95% confidence interval in FOOD ADDITIVE PHOSPHATE (FAP) intake groups for ALL SUBJECTS (WHITE SCALE; 1: n = 66; 2: n = 111, 3: n = 151; 4: n = 109, 5: n = 85; 6: n = 29; p for all 0.097, ANCOVA), for FEMALES (GRAY SCALE; 1: n = 45; 2: n = 77; 3: n = 101; 4: n = 73; 5: n = 55; 6: n = 20; p for all 0.267, ANCOVA), and for MALES (BLACK SCALE; 1: n = 16; 2: n = 39; 3: n = 54; 4: n = 28; 5: n = 33; 6: n = 10; p for all 0.372, ANCOVA).** The data are adjusted for sex (when analyzing all subjects), age, LDL-HDL cholesterol ratio, smoking status, and IMT sonographer class. Statistically significant differences from the highest FAP intake group (ANCOVA) / statistically significant trends (contrast analysis) between FAP intake and IMT are indicated with asterisks (*p < .05, **p < 0.01) (between all other groups p > 0.05).

We report only the results of all subjects and females in the text because among males no significant associations emerged between TP / eTP / FAP intake and IMT (p > 0.05). Results of males are shown in Figures 2, 3 and 4, where relatively large confidence intervals in IMT can be seen. The data were adjusted for sex (when analyzing all subjects), age, LDL-C/HDL-C ratio, smoking status, and IMT sonographer class.

#### *Total phosphorus intake*

Results of ANCOVA are shown in Figure 2. No significant association was present between TP intake and IMT (for all p = 0.233, for females p = 0.293, ANCOVA). However, in between-group comparison in females the 1st and 5th intake quintiles differed significantly (p = 0.035); IMT was higher with higher TP intake. No significant linear trend in associations between TP intake and IMT was observed among all subjects (p = 0.136, contrast analysis), but among females an almost significant positive linear trend was present (p = 0.072, contrast analysis).

#### *Energy-adjusted total phosphorus intake*

Results of ANCOVA are shown in Figure 3. No significant association existed between eTP intake and IMT (for all p = 0.289, for females p = 0.231, ANCOVA). However, in between-group comparison among all subjects the 1st and 5th intake quintiles differed almost significantly (p = 0.064). Among females, the 1st quintile differed almost significantly (p = 0.065) and the 2nd quintile differed significantly (p = 0.034) from the 5th quintile in IMT; IMT was higher with higher eTP intake. Furthermore, a significant positive linear trend occurred between eTP and IMT among all subjects (p = 0.039, contrast analysis), and among females an almost significant positive linear trend (p = 0.067, contrast analysis).

#### *Food additive phosphate intake*

Results of ANCOVA are shown in Figure 4. The population was divided into six groups based on FAP intake scores, as described in the methods section. Between FAP intake and IMT no significant association was observed (for all p = 0.097, for females p = 0.267, ANCOVA). Nevertheless, in between-group analysis among all subjects, IMT was higher with higher FAP intake while the 6th intake group differed significantly in IMT from almost all other groups (1st p = 0.009, 2nd p = 0.032, 3rd p = 0.052, 4th p = 0.006, 5th p = 0.038); this was also the case among females (1st p = 0.037, 2nd p = 0.045, 3rd p = 0.086, 4th p = 0.019, 5th p = 0.113). In addition, a significant positive linear trend existed between FAP intake and IMT among all subjects (p = 0.022, contrast analysis) and among females (p = 0.045, contrast analysis).

## Discussion

Our study demonstrated that dietary phosphorus should be taken into account as a potential cardiovascular risk factor not only in renal patients, but also in the general population. We found positive linear trends in associations among energy-adjusted dietary P intake and FAP intake, and IMT. Moreover, IMT of females was significantly higher in the highest TP intake quintile compared to the lowest intake quintile. The results strengthen the hypothesis that not only elevated serum phosphate concentrations, but also higher dietary P intake may be associated with higher IMT in a normal, healthy population. Data on health effects of high P intake on the normal population are scarce; most of the studies carried out on P intake have focused on bone health [21-24].

We wanted to address our research question separately for TP intake and FAP intake because FAP is more absorbable in the intestine than natural P [7,8]. However, the proportion of FAP in TP is unknown and FAP amounts in foodstuffs between manufacturers vary greatly. We also paid attention to TP intake and, when adjusted for energy intake, describes the P density of the diet, excluding energy intake as a potential confounding factor. While marked variation in TP intake can be seen between the smallest intake quintile (ca. 1100 mg/d) and the highest one (ca. 2300 mg/d), on average all subjects exceeded recommended intakes [4,5]. However, it should be noted that persons in Finland usually have a high dietary Ca intake [2], as also our study subjects had (mean intake about 1200 mg/d).

IMT, the main outcome of the study, is a marker of pre-clinical atherosclerosis and is associated with such traditional CVD risk factors as smoking, diastolic blood pressure, age, serum glucose, and LDL-C concentration [25,26]. Additionally, male sex and black race are associated with higher IMT [25]. Already in younger populations changes in carotid IMT can be seen [27]. FGF-23, a bone-derived hormone-like phosphatonin, has been speculated to participate in the effects of P in CVD, but the mechanisms are unknown [28]. High FGF-23 concentrations have been associated with CVD risk factors [29] and CVD itself [30] but results of many studies are discordant [31], and to our knowledge, no studies have focused on FGF-23 and IMT in the general population. Dietary P load has been shown to increase FGF-23 concentration in serum acutely [32], however, in our study P-FGF-23 concentration was not associated with dietary P intake which may be due to the cross-sectional design and not adjusting for potential covariates.

The regulation of Pi plays a role in vascular calcification. Smooth vascular muscle cells differentiate to osteoblast/chondrocyte-like cells under high Pi concentrations which induce calcification [33,34]. Ruan et al. [12] reported that high S-Pi was associated with higher IMT, and Park et al. [35] found that lower S-Pi was associated with less coronary calcification. However, in our study FAP intake was associated with IMT, but IMT was inversely associated with S-Pi concentrations. It should be noted that analyses on S-Pi were not adjusted for potential covariates, and, in addition, our blood samples were collected when the S-Pi is at its lowest concentration, which may explain the inverse association between FAP intake and S-Pi. Notable is that in the Health Professionals Follow-Up Study, plasma phosphate concentration was not associated with the development of coronary heart disease among males [36]. However, we have no information about the sample collection time of the other studies, and the contradictories may be explained by this. In the studies of Mataix et al. [37] and de Boer et al. [38], dietary P intake correlated only weakly with S-Pi and Mataix et al. [37] concluded that because S-Pi is tightly controlled, dietary P does not affect S-Pi very efficiently. The cross-sectionality of these studies and our study may affect the results because clinical studies carried out at our unit indicate an acute dose–response effect of dietary P on S-Pi concentration [8,21,22].

One limitation of the study is its cross-sectional design, which precludes an evaluation of causality. However, it is also difficult to know if the dietary intake methods in the current study are indicative of usual diet. Selection bias may also exist; our study subjects may be healthier than the normal population because they were interested in participating in this kind of study. There may be some differences between the sexes in adaptation to P; here the association was affected by sex. Women being more vulnerable to P, especially through Ca and PTH metabolism [39], may explain not finding differences among males. Nevertheless, the distribution of sexes in our study was not equal; the number of females was twice the number of males, and the sizes of FAP groups differed widely, with the smallest group sizes having the highest FAP intake scores. In the case of males, the size was only ten persons, which may also cause the lack of power in the analyses.

Strengths of our study are that many biomarkers were analyzed and extensive background data were available. In large studies where the main focus was not nutritional, food intake data may have been collected less accurately than in our study. The way that FAP intake is calculated may be criticized, but one should realize that the information on the exact amounts of FAP in foodstuffs is not available.

## Conclusion

Our results show significant linear trends in the associations among energy-adjusted dietary phosphorus intake, food additive phosphorus intake and carotid intima-media thickness in a healthy, middle-aged Caucasian population. Based on these results, high dietary P intake should be further investigated due to its potential association with CVD risk factors in the general population, not only in renal patients. Furthermore, prospective, or even better, long-term intervention studies are required to evaluate the possible impact of dietary P burden on risk of CVD.

## Competing interest

All authors state that they have no conflicts of interest.

## Authors’ contribution

STI, HJK, VEK, and CJELA designed the study. MIT designed the IMT measurement. STI, HJK, VEK, EMS, MHP, and MUMK collected the data. STI and EMK analyzed the data. STI, CJELA, and EMK interpreted the data. STI drafted the manuscript with help of CJELA. STI, EMK, and CJELA are responsible for the integrity of data analysis. All authors read, reviewed and approved the final manuscript.
